# Economic evaluation of implementation science outcomes in low- and middle-income countries: a scoping review

**DOI:** 10.1186/s13012-022-01248-x

**Published:** 2022-11-16

**Authors:** Akash Malhotra, Ryan R. Thompson, Faith Kagoya, Felix Masiye, Peter Mbewe, Mosepele Mosepele, Jane Phiri, Jairos Sambo, Abigail Barker, Drew B. Cameron, Victor G. Davila-Roman, William Effah, Brian Hutchinson, Michael Laxy, Brad Newsome, David Watkins, Hojoon Sohn, David W. Dowdy

**Affiliations:** 1 Department of Epidemiology, Johns Hopkins Bloomberg School of Public Health, Baltimore, MD USA; 2 Infectious Diseases Research Collaboration, Kampala, Uganda; 3 University of Zambia, Lusaka, Zambia; 4 Centre for Infectious Disease Research in Zambia, Lusaka, Zambia; 5 University of Botswana, Gaborone, Botswana; 6 Ezintsha, Faculty of Health Sciences, University of Witwatersrand, Johannesburg, South Africa; 7 Cavendish University Zambia, Lusaka, Zambia; 8 Washington University in Saint Louis, Saint Louis, MO USA; 9 Department of Health Policy and Management, Yale School of Public Health, New Haven, CT USA; 10 Center for Global Noncommunicable Diseases, RTI International, Seattle, WA USA; 11 Technical University of Munich, Munich, Germany; 12 Fogarty International Center (FIC), National Institutes of Health (NIH), Bethesda, MD USA; 13 University of Washington, Seattle, WA USA; 14 Department of Preventive Medicine, Seoul National University College of Medicine, Seoul, South Korea

**Keywords:** Economic evaluation, Implementation science, Implementation outcomes, Cost-effectiveness, Low- and middle-income countries, Infectious disease, Scoping review

## Abstract

**Background:**

Historically, the focus of cost-effectiveness analyses has been on the costs to operate and deliver interventions after their initial design and launch. The costs related to design and implementation of interventions have often been omitted. Ignoring these costs leads to an underestimation of the true price of interventions and biases economic analyses toward favoring new interventions. This is especially true in low- and middle-income countries (LMICs), where implementation may require substantial up-front investment. This scoping review was conducted to explore the topics, depth, and availability of scientific literature on integrating implementation science into economic evaluations of health interventions in LMICs.

**Methods:**

We searched Web of Science and PubMed for papers published between January 1, 2010, and December 31, 2021, that included components of both implementation science and economic evaluation. Studies from LMICs were prioritized for review, but papers from high-income countries were included if their methodology/findings were relevant to LMIC settings.

**Results:**

Six thousand nine hundred eighty-six studies were screened, of which 55 were included in full-text review and 23 selected for inclusion and data extraction. Most papers were theoretical, though some focused on a single disease or disease subset, including: mental health (*n* = 5), HIV (*n* = 3), tuberculosis (*n* = 3), and diabetes (*n* = 2). Manuscripts included a mix of methodology papers, empirical studies, and other (e.g., narrative) reviews. Authorship of the included literature was skewed toward high-income settings, with 22 of the 23 papers featuring first and senior authors from high-income countries. Of nine empirical studies included, no consistent implementation cost outcomes were measured, and only four could be mapped to an existing costing or implementation framework. There was also substantial heterogeneity across studies in how implementation costs were defined, and the methods used to collect them.

**Conclusion:**

A sparse but growing literature explores the intersection of implementation science and economic evaluation. Key needs include more research in LMICs, greater consensus on the definition of implementation costs, standardized methods to collect such costs, and identifying outcomes of greatest relevance. Addressing these gaps will result in stronger links between implementation science and economic evaluation and will create more robust and accurate estimates of intervention costs.

**Trial registration:**

The protocol for this manuscript was published on the Open Science Framework. It is available at: https://osf.io/ms5fa/ (DOI: 10.17605/OSF.IO/32EPJ).

**Supplementary Information:**

The online version contains supplementary material available at 10.1186/s13012-022-01248-x.

Contributions to the literature

- Health economics and implementation science are used to inform health policy. However, the two fields have historically not been integrated, with economic evaluations often omitting implementation costs.
- This scoping review summarizes the existing literature on integrating implementation costs into economic analyses. This literature is growing rapidly but is still focused on high-income countries, and there was large heterogeneity in how implementation costs were defined and collected.
- We outline steps to improve integration of implementation into economic evaluation, including a more precise definition of implementation costs, appropriate timing for implementation cost collection, and distinguishing implementation costs from operational expenses.

## Background

Economic evaluation is widely used to help decision-makers evaluate the value-for-money tradeoffs of a variety of public health interventions [1]. Only recently, however, have the fields of economic evaluation and implementation science begun to synergize. In most existing guidelines for the conduct of economic evaluations (including cost-effectiveness analyses), little attention is paid to program implementation and improvement—such as costing the design of health interventions (e.g., focus groups to design intervention, sensitization events), their initial implementation (e.g., development of infrastructure, hiring and training of program staff), and sustainability (e.g., annual re-trainings of personnel) [2, 3]. As a result, most cost-effectiveness analyses to date have focused on estimating the price to deliver an intervention after it has already been designed and launched, underestimating their total cost [4–6].

While germane to high-income countries, the costs of initial design, implementation, and improvement are particularly salient in low- and middle-income countries (LMICs)—where resources are often constrained, and initial implementation of health-related interventions may require the establishment of new infrastructure (e.g., information technology, transportation systems) and/or technical assistance from consultants whose salaries often reflect prevailing wages in higher-income settings. As a result, economic evaluations that do not consider implementation costs beyond the costs of delivery will underestimate the total cost of interventions. Omission can mislead decision-makers about program feasibility—by glossing over up-front investments and resources that are required to design and launch a program, and recurring costs to maintain it. It may also miss an opportunity to highlight infrastructural investments (e.g., development of health information systems) with transformative potential for other priorities—economies of scope being a criterion that some decision-makers may consider for investments.

The growing field of implementation science represents an opportunity to help fill this knowledge gap [7]. Many conceptual frameworks for implementation science recommend collecting costs and economic data alongside other implementation outcomes [8–10]. Despite these general recommendations, specific guidance is lacking as to how economic data should be collected in the context of implementation or how to interpret such data to inform decision-making. There is a need for more practical exploration of how implementation frameworks can inform economic evaluations such as cost-effectiveness analyses and budget impact analyses.

To date, little is known about the scope of research that has been performed at the intersection of economic evaluation and implementation science, particularly in LMICs. We therefore conducted a scoping review to investigate the availability, breadth, and consistency of literature on the integration of economic evaluation and implementation science for health interventions in LMICs and to identify gaps in knowledge that should be filled as the fields of economic evaluation and implementation science are increasingly integrated.

## Methods

The literature search strategy was created following guidelines from the Joanna Briggs Institute (JBI) Manual for Evidence Synthesis [11] and the PRISMA-ScR checklist [12]. The protocol for this scoping review was published online May 2, 2022 and is available from the Open Science Framework at: https://osf.io/ms5fa/ (DOI: 10.17605/OSF.IO/32EPJ). The protocol is also included as supplementary material for this manuscript.

**Fig. 1:**
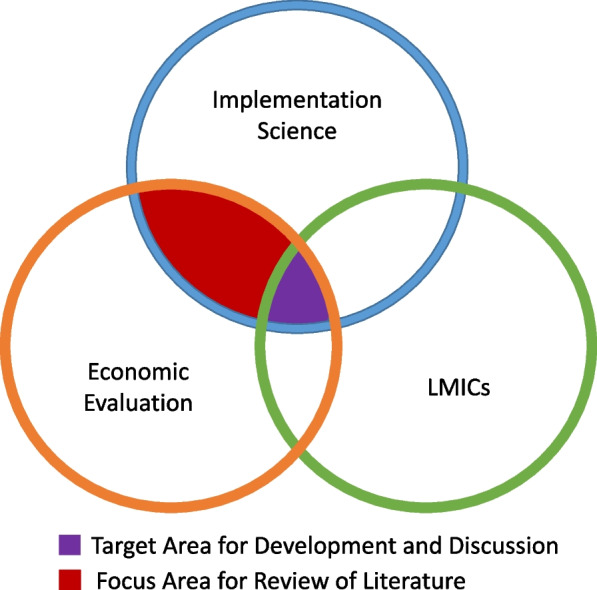
Scope of review. We sought primarily to identify articles that evaluated the integration of implementation science and economic evaluation in the context of low- and middle-income countries (LMICs) [purple shaded area]. To better inform this space, we also included articles from high-income settings [red shaded area]. Both theoretical and empirical studies were included in this review

The primary question/objective of this review was: “What is the scope of the existing scientific literature on integration of implementation science and economic evaluation for health interventions in LMICs?” (Fig. 1).

### Eligibility criteria

Eligible papers must have been written in English, Spanish, French, or Portuguese, and were required to focus on studies of programs or policies that emphasize targeting of health interventions. Included papers were required to address components of both implementation science and economic evaluation. We included methodology papers, review papers, peer-reviewed clinical research, grey literature, and conference abstracts, and allowed studies that used both empirical data and theoretical frameworks. Protocol papers were excluded. No restrictions were placed on settings or study populations, but priority was given to studies centered in LMICs. Studies from high-income countries (HICs) were eligible if their methodology and findings were deemed by both independent reviewers to be potentially relevant and applicable to LMICs. As an example of a method that was deemed to be potentially relevant to LMICs, Saldana et al. (2014) mapped implementation resources and costs in the implementation process for a Multidimensional Foster Care Intervention in the USA; this mapping process could be replicated for other diseases in the LMIC context [13].

### Information sources

We searched Web of Science and PubMed for papers from January 1, 2010 through December 31, 2021. We also referred to the Reference Case of the Global Health Costing Consortium and searched grey literature using the methodology outlined by the Canadian Agency for Drugs and Technology in Health [14, 15]. We used a mix of Medical Subject Headings (MeSH terms) and search queries in English related to economic evaluation and implementation science, trying different combinations of phrases to capture as many articles as possible. The full list of MeSH and search terms is available in the Additional file 1. The PubMed and Web of Science search engines sort articles in order of relevance. Due to capacities on human resources, we aimed to screen about the first 3000 articles that were listed based on relevancy from each database. To find additional papers for abstract screening, after completing the database searches, we reviewed the references of all identified articles (“backward snowballing”) as well as lists of publications that cited the included articles (“forward snowballing”). We also reviewed all publications by any author who had two or more first/senior-author papers in the final publication list. The snowballing and author searches were done using Google Scholar.

### Selection of sources of evidence

After completion of the initial literature search, all articles were screened for eligibility and inclusion. The screening process was done in two stages. First, the titles and abstracts of all studies were independently reviewed by two authors (AM and RRT) for eligibility. For title screening, we excluded papers which had no term related to economic evaluation or implementation science. For abstract screening we excluded papers which had no term related to implementation in their abstracts. Both authors voted independently on whether to include the study, and if deemed ineligible, a reason for exclusion was provided. Any conflicts were resolved by discussion with a third reviewer (DWD).

After the abstract screening, all studies that received “eligible/include” votes from both reviewers underwent a full-text screening of the entire publication. The same two authors independently reviewed each publication for eligibility, with conflicts resolved by the same third author. For full-text screening, we removed papers that did not directly discuss or explore implementation science and economic outcomes, or that did not otherwise meet the eligibility criteria described above. After both rounds of screening, remaining studies were subject to data extraction (using a standardized abstraction tool), final analysis, and evaluation.

### Data charting and data items

We created a data extraction tool in Microsoft Excel to guide capture of relevant details from included articles. The form was piloted before use to ensure all fields of interest were captured. Data charting for all papers was completed in duplicate, separately, by two authors. Extraction focused on metadata, study details, interventions used, frameworks considered, methodology, results and outcomes, and key takeaways. The full list of information extracted from studies is available in the Additional file 1. Results were synthesized using both quantitative and qualitative methods—including enumeration of key concepts, perspectives, populations, and themes.

## Results

**Fig. 2:**
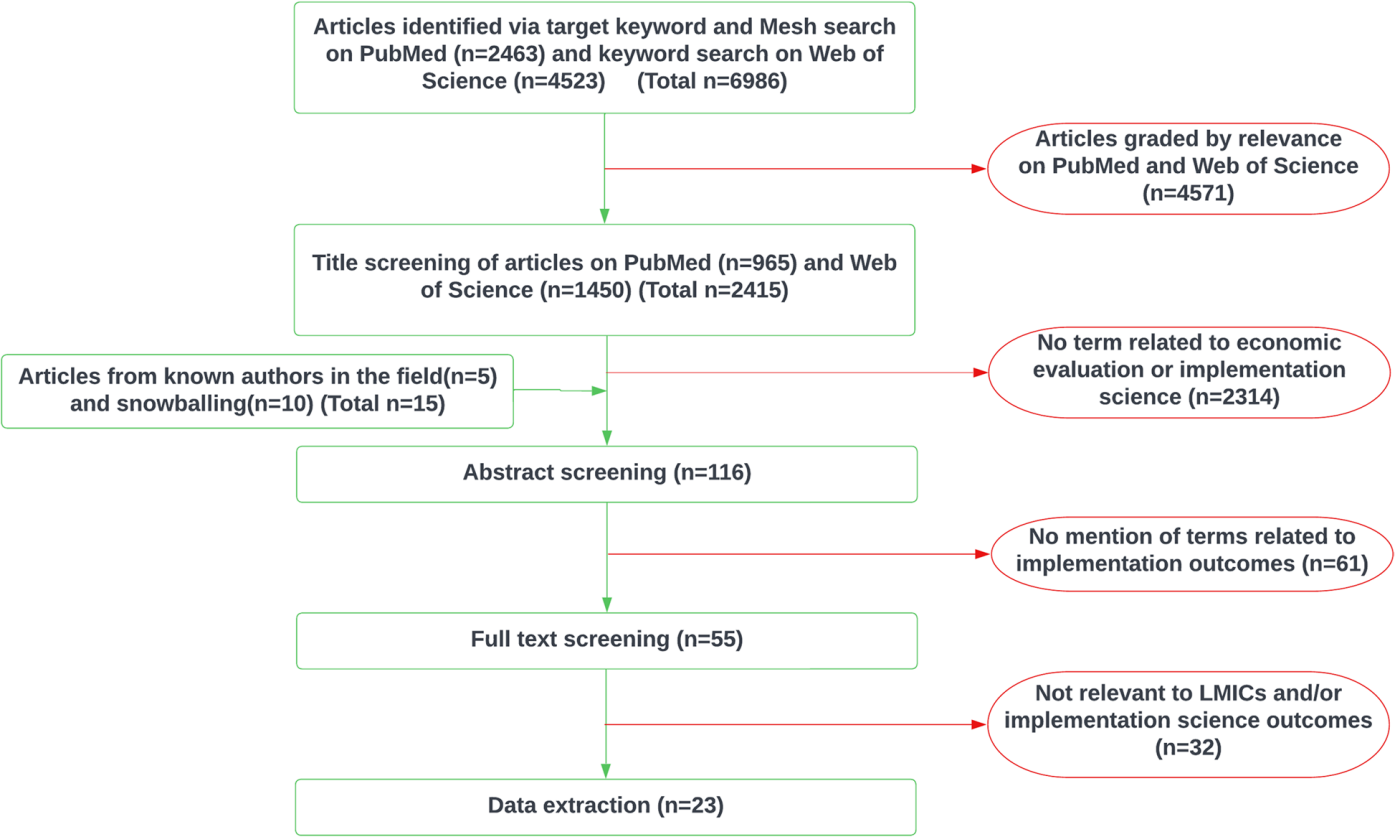
PRISMA flow diagram. Rectangular boxes outlined in green refer to the articles included at each step of the review. Curved boxes outlined in red list reasons for excluding articles from the review at each step. The PubMed and Web of Science search engines list articles in order of relevance. Articles that feature low on the list (4571/6986, 65%) were excluded. Following this step, 2314 of 2415 (96%) remaining articles were excluded for not having terms related to economic evaluation or implementation science, 61 of 116 articles (53%) screened by abstract were excluded for not having implementation outcomes, and 32 of 55 articles (58%) screened by full text were excluded for not being relevant to LMICs and/or implementation science outcomes

From an initial list of over 6,900 articles, we identified 23 unique articles for data extraction (Fig. 2).

Characteristics of included studies are presented in Table 1. Despite our explicit focus on LMICs, only one of 23 papers (4%) had a first or senior author from an LMIC [16]. Of the nine studies that provide empirical results, only one (11%) focused on an LMIC [17], five studies (55%) focused on high-income settings [13, 18–21], one (11%) looked at both LMICs and HICs [22], and two (22%) had a global focus [16, 23]. Six (26%) of the 23 papers focused on infectious diseases [17, 22, 24–27], five (22%) on mental health [13, 19, 28–30], and three (13%) on non-infectious diseases [18, 20, 21]. The other studies did not empirically focus on one specific disease area. For example, Cidav et al. (2020) advanced a theoretical framework to incorporate cost estimates into implementation evaluation [23]. Most research on this subject was recent: 12 of the 23 studies were published during the last 2 years of the review [17, 18, 22–25, 27–32].

**Table 1:** Metadata and other characteristics of included studies

Characteristic	***n***/***N*** (%)	References
**Region of origin of first author** (place of work)		
Africa	0 (0%)	–
Asia	0 (0%)	–
Europe, North America, Australia	22 (96%)	[13, 17–37]
Latin America	1 (4%)	[16]
**Region of origin of senior author** (place of work)		
Africa	0 (0%)	–
Asia	0 (0%)	–
Europe, North America, Australia	22 (96%)	[13, 17–37]
Latin America	1 (4%)	[16]
**Region of research (for empirical or case studies only)**		
Africa	2 (22%)	[17, 22]
Asia	1 (11%)	[22]
Europe, North America, Australia	6 (67%)	[13, 18–22]
Latin America	0 (0%)	-
Global	2 (22%)	[16, 23]
**Disease/focus area**		
Mental health	5 (22%)	[13, 19, 28–30]
Diabetes	2 (9%)	[20, 21]
HIV/AIDS	3 (13%)	[17, 25, 26]
Tuberculosis	3 (13%)	[22, 24, 27]
Universally applicable^a^	9 (39%)	[16, 23, 31–37]
All other^b^	3 (13%)	[18, 29, 36]
**Publication date**		
2020 and 2021	12 (52%)	[17, 18, 22–25, 27–32]
2016 to 2019	8 (35%)	[16, 19, 21, 26, 33–36]
2011 to 2015	3 (13%)	[13, 20, 37]
**Study design**		
Review	7 (30%)	[25, 30–35]
Methodology	9 (39%)	[16, 20, 23–25, 28, 29, 36, 37]
Empirical research	9 (39%)	[13, 16–23]
All other	2 (9%)	[26, 27]
**Economic perspective**		
Healthcare system (provider)	10 (43%)	[13, 16–19, 21–24, 29]
Patient	0 (0%)	–
Societal	12 (52%)	[20, 25, 27, 28, 30–37]
Not described/not applicable	1 (4%)	[26]
**Linkage to implementation science or costing framework**^c^		
Yes	13 (57%)	[13, 16, 20, 23–28, 31–33, 35]
No	10 (43%)	[17–19, 21, 22, 29, 30, 34, 36, 37]

^a^The methods discussed are not being applied to a specific disease

^b^Includes stroke, heart disease, and one purely theoretical paper

^c^The papers explicitly mention the use of a costing or implementation framework

Seven (30%) of the 23 studies were reviews (25,30–35), nine (39%) were methodologically focused [16, 20, 23–25, 28, 29, 36, 37], and nine (39%) used empirical data [13, 16–23]. A few papers had multiple types of study design.

Of the nine empirical studies, four (44%) could be mapped to an existing costing or implementation framework. Cidav et al. (2020) leveraged Proctor’s Outcomes for Implementation Research Framework to systematically estimate costs [23], Hoomans et al. (2011) provided details of a total net benefits approach [20], Saldana et al. (2014) leveraged the Stages of Implementation Completion (SIC) template for mapping costs [13], and da Silva Etges et al. (2019) presented a Time Driven Activity Based Costing (TDABC) framework [16]. A few other frameworks such as the Consolidated Framework for Implementation Research [9], Implementation Outcome Framework [8], Policy Implementation Determinants Framework [38], and the RE-AIM Framework [39], were applied by other included studies.

Papers covered a variety of focus areas, ranging from costing methodologies [16, 20, 23–25, 28, 29, 36, 37], and determinants of implementation [31]*,* to reviews highlighting the paucity of evidence using economic evaluation in implementation science [34]. As examples of topic areas, Hoomans and Severens (2014) mentioned the lack of a widely accepted mechanism to incorporate cost considerations into implementation of programmatic guidelines [37]. Bozzani et al. (2021) discussed integration of “real-world” considerations to more accurately estimate the true cost of an intervention [27]. Nichols and colleagues (2020) argued that many implementation costs are not actually one-off payments, as they are typically treated in economic analyses [17]. Salomon et al. (2019) discussed how favorable outcomes of most cost-effectiveness analyses may be due to a systematic bias leading to an underestimation of costs or an overestimation of impact [26].

In terms of developing frameworks for costing implementation of health interventions, Cidav et al. (2020) provided the breakdown of costs both by the implementation strategy and the phase of implementation [23]. Krebs and Nosyk (2021) discussed mapping intervention costs to implementation outcomes like maintenance [25]. Sohn et al. (2020) proposed partitioning an intervention into three phases (design, initiation, and maintenance) to measure costs at different timepoints in the implementation process [24]. Of the few empirical studies included, outcomes centered around either cost per patient, cost per participant, or the net and marginal cost of implementation. Measured implementation cost outcomes ranged from simple collection of training costs at study launch [22] to detailed collection of data on installation, maintenance, and personnel costs across several months or years of follow-up [17, 24]. Many studies did not offer an explicit definition of implementation costs, nor did they provide specific context to what goods or services would be included.

## Discussion

This scoping review analyzed 23 articles evaluating implementation outcomes and implementation costs in the health economic literature. Our review showed that there is a growing literature on implementation costs and methodologies for evaluating implementation costs. However, among the reviewed articles, there is large heterogeneity in what is meant by implementation costs, and the relevance of the current literature to LMICs is weak. Only 8 of the 23 articles were based in LMICs or directly alluded to the applicability of their techniques in low-resource settings—and only one had a first or senior author from an LMIC setting. This gap reflects trends in economic evaluation in general, with only a small portion of all costing analyses occurring in LMICs [26]. The broad methodologies and concepts for collecting costs may be translatable, in principle, from high- to low-income settings. However, owing to their richness of available costing data and large budgets, many studies and techniques from high-income settings may not be applicable or realistic in low-income settings [18, 19, 21]. Since implementation and/or improvement costs may represent a larger portion of the total cost of interventions in LMICs, the paucity of both economic evaluations and implementation costing studies from LMICs represents an important area for future research.

An important component of this research is to evaluate the feasibility of sustaining these health interventions and bringing them to scale—and the “sunk” costs to the health system if such scaling-up does not succeed. The importance of costing the sustainability of interventions is especially pertinent in LMICs, where high personnel turnover and sub-optimal infrastructure can make maintenance/sustainability particularly expensive. To capture such costs, researchers should consider incorporating cost items such as equipment breakdown (e.g., parts and labor to repair equipment), hiring and training of replacement staff, quality assurance and control, and investments in underlying infrastructure (e.g., stable electrical supply) that may be required to sustain health interventions in LMIC contexts.

Cultural adaptation is another critical component of successful implementation in the LMIC context. Such adaptation—often including such steps as formative qualitative research, application of contextual frameworks, and human-centered design (HCD)—is often very expensive relative to delivery of the intervention itself. For example, implementation of even apparently simple interventions such as umbilical chlorhexidine may require qualitative research, ethnographic inquiry, and community engagement—often at great cost [40, 41]. As another illustrative example, the estimated cost of HCD for a tuberculosis contact investigation strategy was $356,000, versus a delivery cost of $0.41 per client reached [42].

We note that “implementation” and “implementation science” are terms used in one specific field of research; other fields may use different terms, such as “improvement.” While this scoping review used “implementation” as a grounding term, use of “improvement” and “improvement science” may have resulted in different findings. For example, McDonald et al. [43] synthesized evidence to better explain the quality improvement field for practitioners and researchers, and Hannan et al. [44] studied the application of improvement science to the field of education. In evaluating the integration of costs, therefore, future studies in the field of implementation science may also wish to draw on literature that does not center on the term “implementation.”

**Fig. 3:**
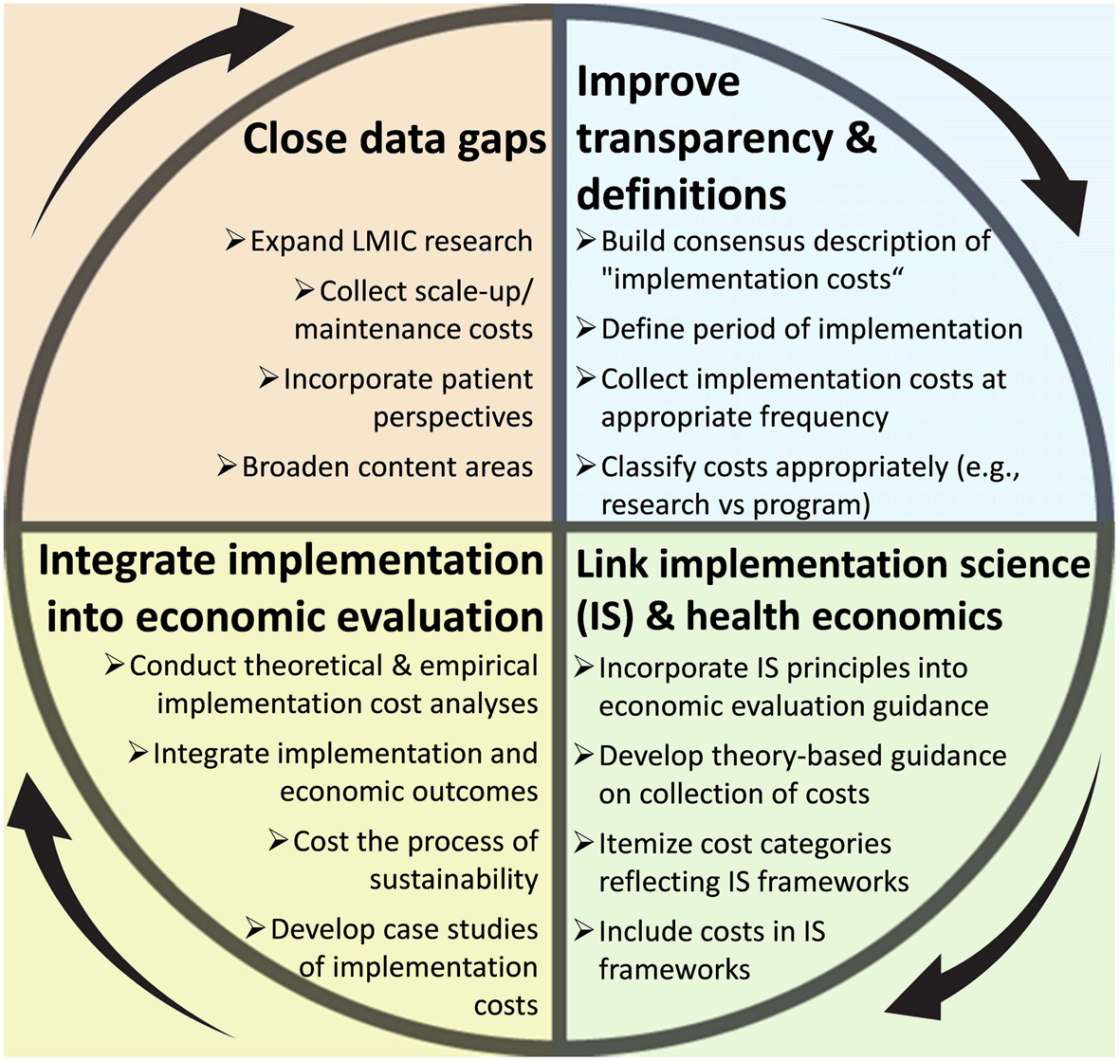
Framework for improving implementation costing in LMICs. Each quadrant represents a key knowledge domain that, if addressed, will improve our understanding of implementation costs in LMICs. Arrows illustrate that these domains are cyclical and interdependent, such that addressing one domain will help to refine questions in the next

To improve transparency and consistency in the definition and collection of implementation costs, at least four steps may be useful (Fig. 3). First, implementation costing studies could explicitly define the period of the implementation process during which costs are being collected. Both health economists and implementation scientists have highlighted the importance of defining the time of the evaluation [24, 45]. Instinctually, the general line of thought may be that “implementation” is tied to the beginning (especially) and middle of a process. But, implementation is “the process of making something active or effective”: a process which may have no end point given the need for continuous acquisition or development of resources to facilitate program upkeep (e.g., training new cohorts of health professionals) and monitoring and evaluation to ensure efficient use of program resources and evaluate impact. One suggested approach to delineating [24, 45] the timing of implementation costs includes three phases: “design/pre-implementation”, “initiation/implementation”, and “maintenance/post-implementation”. Second, as argued by Nichols (2020), authors should note if implementation costs are incurred and collected at a single point in time, or on a recurring basis [17]. Third, the activities and items that are included as “implementation costs” should be made more explicit. Researchers should state what types of activities, materials, and goods are included in their implementation costs, and align on development of methods to capture cost estimates. In doing so, consensus can be developed as to a reasonable taxonomy of implementation costs in health interventions. Finally, consensus should be developed regarding what constitutes the implementation process itself. Many studies, for example, included routine operational and delivery costs among the costs of “implementing” an intervention. Although such costs are critical to the implementation of health interventions, including operational expenses as “implementation” costs may have the unintended consequence of ignoring other costs required for design, initiation, and sustainability of interventions. A similar distinction needs to be made to separate research costs, such as Institutional Review Board approval, from implementation expenses, as the two are often conflated. Clearer consensus—with more examples—of incorporating such costs as separate from those of routine operation and delivery could lead to more frequent inclusion of these costs in economic evaluations.

A second priority for evaluating implementation costs is to strengthen the emerging linkage between the fields of economic evaluation and implementation science. Conceptual frameworks in the implementation science literature can be useful for informing economic evaluations – but these frameworks are rarely used for that purpose. Examples of frameworks that do explicitly include costs include an 8-step framework by da Silva Etges et al. [16] to apply time-driven activity-based costing (TDABC) in micro-costing studies for healthcare organizations, where resources and costs for each department and activity are mapped to calculate the per patient costs and perform other costing analyses. As another example, Cidav et al. [23] combine the TDABC method with the implementation science framework by Proctor et al. [8] to clearly map the implementation process by specifying components of the implementation strategy and assigning costs to each action as part of the strategy. Implementation science frameworks that explicitly include costs as an outcome should consider how these costs can and should be collected—and inclusion of economic outcomes should be prioritized in the development of “next-generation” implementation science frameworks. As with the definition of implementation costs itself (as discussed above), these frameworks should include guidance regarding the types of activities and costs that researchers should collect, and appropriate methodologies for collecting those economic data. Such linkages should also be bi-directional; implementation frameworks can draw on the wealth of empirical costing studies to inform such recommendations. In doing so, closer communications between experts in implementation science and experts in economic evaluation will be essential.

Similarly, economic evaluations should increasingly seek to integrate implementation outcomes. Many economic evaluations assume perfect implementation or make overly optimistic assumptions about intervention uptake, without considering the real-world programmatic costs required to achieve that level of uptake. Several of the papers included in this review stressed the importance of acknowledging this disconnect, arguing it can bias studies toward favorable outcomes and unrealistic estimates of both impact and cost [26, 27, 32]. The need for incorporating implementation outcomes into economic studies extends to theory as well. Krebs and Nosyk (2021), for example, showed how the scale of delivery for an intervention can be estimated using reach and adoption, and that the “realistic” scale of delivery is much lower than the “ideal/perfect” situation usually assumed in economic analyses [25]. By better defining how costs should be collected within implementation science frameworks and by integrating implementation outcomes into economic analyses, researchers can perform more standardized, accurate, comparable, and programmatically viable economic evaluations.

As with any study, our work has certain limitations. First, as a scoping (rather than systematic) review, our search was not as structured or comprehensive as a formal systematic review [46]. As a scoping review, we also did not formally assess the quality of included manuscripts. Second, while this field of research is expanding, the literature on this topic was sparse, and the extracted data were heterogeneous—making comparisons across individual manuscripts difficult in many cases. This heterogeneity, while a weakness in the corresponding literature, represents an important finding of this review—and a key area of focus for future research. A third limitation is that we did not include studies published beyond 2021. This is a rapidly growing area of research, and this review will therefore need frequent updating. Finally, though we did allow grey literature to be included, we did not explicitly search any databases, repositories, or websites specific to the grey literature. This could lead to underrepresentation of this information in the review and discussion, and limit our findings relevant to policy implications.

## Conclusion

In summary, this scoping review of 23 studies at the interface of economic evaluation and implementation science revealed that this literature is sparse (but rapidly growing), with poor representation of LMIC settings. This literature was characterized by heterogeneity in the considered scope of implementation costs—speaking to the importance of developing consensus on the activities and costs that should be considered as “implementation costs”, being explicit regarding the timing of those costs (both timing of incurring and evaluating costs), and more clearly distinguishing between implementation and operational costs (so as not to implicitly exclude implementation costs). These studies also highlighted the importance—and the opportunity—of forging closer linkages between the fields of implementation science and economic evaluation, including formal collaborations between experts in both fields. Closer integration of implementation science and economic evaluation will improve the relevance of economic studies of implementing health interventions, leading to more programmatically useful and robust estimates of the costs of interventions as implemented in real-world settings.

## Supplementary Information

**Additional file 1.** Protocol for scoping review.

**Additional file 2.** Preferred Reporting Items for Systematic reviews and Meta-Analyses extension for Scoping Reviews (PRISMA-ScR) Checklist.

## Data Availability

The extraction tool and all abstracted data are available from the authors upon reasonable request.

## Abbreviations

LMIC: Low- and middle-income country
RE-AIM: Reach, Effectiveness, Adoption, Implementation, Maintenance
JBI: Joanna-Briggs Institute
Prisma-ScR: Preferred Reporting Items for Systematic Reviews and Meta-Analyses, Scoping Review
MeSH: Medical Subject Heading
HIV: Human immunodeficiency virus
HCD: Human-Centered Design
TDABC: Time-driven activity-based costing
