# Supplementary material for: Economic evaluation of implementation science outcomes in low- and middle-income countries: a scoping review
Source: Implement Sci. 2022 Nov 16;17:76. doi: 10.1186/s13012-022-01248-x (PMC9670396; doi:10.1186/s13012-022-01248-x)
Supplement: Supplementary file 1 — Additional file 1. Protocol for scoping review. [file 13012_2022_1248_MOESM1_ESM.docx]

TITLE

Economic evaluations of implementation science outcomes in low-and-middle-income settings: a scoping review protocol

INTRODUCTION

Health economics, or economic evaluation, has gained traction in public health research over the past few decades. Health economists use economic theories regarding efficiency, productivity, competition, and pricing to develop informed estimates on which health interventions are the most efficient, equitable, and cost-effective. Health economics is used to help policymakers and governments decide what health interventions to prioritize and build budget estimates based on the cost of supporting various public health programs and practices. The Consolidated Health Economic Evaluation Reporting Standards (CHEERS) statement is the primary guideline for conducting and reporting economic evaluations,^1,2^ especially in high-income countries (HICs). The CHEERS guidelines consist of a 28-item standardized checklist for researchers to ensure economic studies are reliable, useful, and interpretable by readers and policymakers. In low- and middle-income countries (LMICs), beyond the CHEERS guidelines there are additional LMIC-specific guidance documents, and an increased emphasis on reference cases.^3–6^

Globally, there has been a strong push over the past 20 years to introduce implementation science into public health research. In public health, implementation science broadly refers to the study of methods and strategies to facilitate the uptake of evidence-based interventions and practices into regular, or “real-world”, use by policymakers and health practitioners. One of the first frameworks for evaluating public health interventions from an implementation science perspective was the RE-AIM framework,^7^ which drew attention to the reach and representativeness of the settings and populations in which clinical research is conducted.

Since the release of the RE-AIM framework, numerous additional frameworks have been proposed. One of the best-known is by Proctor et al (2011) that introduced distinct “implementation outcomes” to measure the overall success and feasibility of implementing a clinical intervention.^8^ Numerous implementation frameworks, including Proctor et al, specifically mention collecting estimates of cost as an implementation outcome. However, these frameworks rarely provide information or methodology recommendations to researchers on how best to collect this information. Without this guidance, there is large heterogeneity in the methods used by public health researchers to collect implementation-specific economic information. This leads to large variability in the quality of economic implementation data collected and can make it difficult to compare economic outcomes across different studies and interventions.

This scoping review will be conducted to evaluate the existing literature on implementation science and economic evaluation in high-income countries (HIC) and low-and-middle income countries (LMICs) to identify key methodology papers and empiric examples for the integration of implementation science and economic evaluation. Using this information, we aim to build a comprehensive framework for the economic evaluation of implementation outcomes, focusing on LMICs. The framework will identify best practices for collecting economic information on various stages of the implementation process, ways to integrate implementation outcomes into economic modeling and uncertainty estimates, and ways to validate implementation costs. A preliminary search for existing scoping and systematic reviews of the subject has been conducted, focusing on the Pubmed and Web of Science databases for January 2010 – December 2021.

The objective of this scoping review is to investigate the availability, breadth, and consistency of literature on the integration of economic evaluation and implementation science for health interventions in low-and-middle income countries.

# **METHODS**

## STUDY DESIGN

Our scoping review is adapted from the Joanna Briggs Institute (JBI) Manual for Evidence Synthesis.^9^ The corresponding PRISMA-ScR checklist will be used for writing and reporting the final results of this scoping review in a peer-reviewed manuscript.^10^ The scoping review protocol, as per the JBI Manual, involves the description of the following components:

1. Title
2. Introduction
3. Scoping review question
4. Search strategy
5. Inclusion criteria
6. Source of evidence selection
7. Data extraction
8. Analysis of the evidence
9. Presentation of the results

## SCOPING REVIEW QUESTION

The scoping question we are seeking to answer through this review is “What frameworks and examples exist in the literature on how frameworks of implementation science are incorporated into efforts of economic evaluation and how frameworks of economic evaluation are incorporated into efforts of implementation science in health studies in low-and-middle-income settings?” The specific sub-questions are as follows:

1. What is the theoretical literature available discussing economic evaluation in implementation science outcomes?
2. What is the empirical literature available discussing economic evaluation in implementation science outcomes?
3. What is the theoretical literature available discussing economic evaluation and implementation science outcomes in low-and-middle-income countries?
4. What is the empirical literature available discussing economic evaluation and implementation science outcomes in low-and-middle-income countries?
5. What are the frameworks guiding measurement of implementation science outcomes? Do these frameworks provide methodologies for integration with economic evaluation?
6. What are the knowledge gaps in the synthesis of economic evaluation and implementation science outcomes?
7. What methods are currently used to integrate implementation science outcomes into models and calculations of economic outcomes?
8. How is implementation science conceptualized in economic evaluations of health interventions?


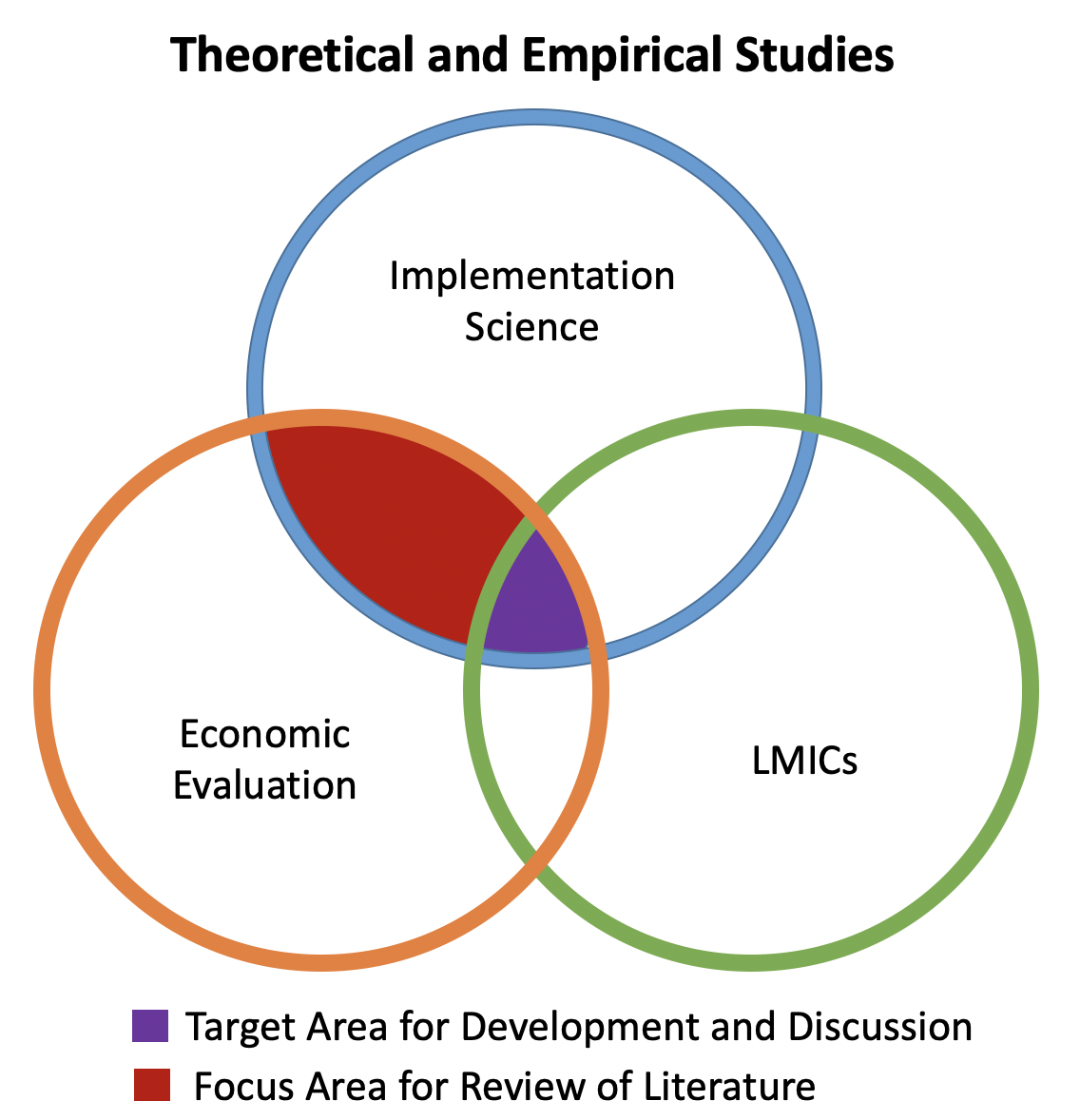


Figure 1. Target areas for the literature review (red) and for in-depth assessment/discussion (purple).

## SEARCH STRATEGY

We will search the following databases for identification of relevant literature: Web of Science, Pubmed, and Google Scholar from January 2010 to July 2021. We will also refer to the Reference Case of the Global Health Costing Consortium and search the gray literature using the methodology outlined by the Canadian Agency for Drugs and Technology in Health.^11^

The search strategy will constitute the use of both Medical Subject Headings (MeSH) and free text entry search queries related to implementation science and economic evaluation. Since literature on this topic is anticipated to be limited, we will apply a combination of search strategies and terms to shortlist relevant research articles. Keywords used in combination will include “economic evaluation”, “health economics”, “cost-effectiveness”, and “implementation science” Eligible papers will be written in English, Spanish, French, or Portuguese, with additional efforts to include literature in other languages when possible.

During the review we will first perform a preliminary screening of the title and abstract. Based on this initial screening we will prioritize articles and perform full text screening in the specified order. While conducting the full text screening, we will also scan the titles and abstracts of articles listed in the references to ensure we are comprehensive with our search and able to identify other relevant literature. We will also run focused searches for additional publications from all authors of included articles.

The shortlisted articles will be stored on a secure OneDrive cloud folder and managed using Mendeley, which will be accessed by the team members for further screening and data extraction.


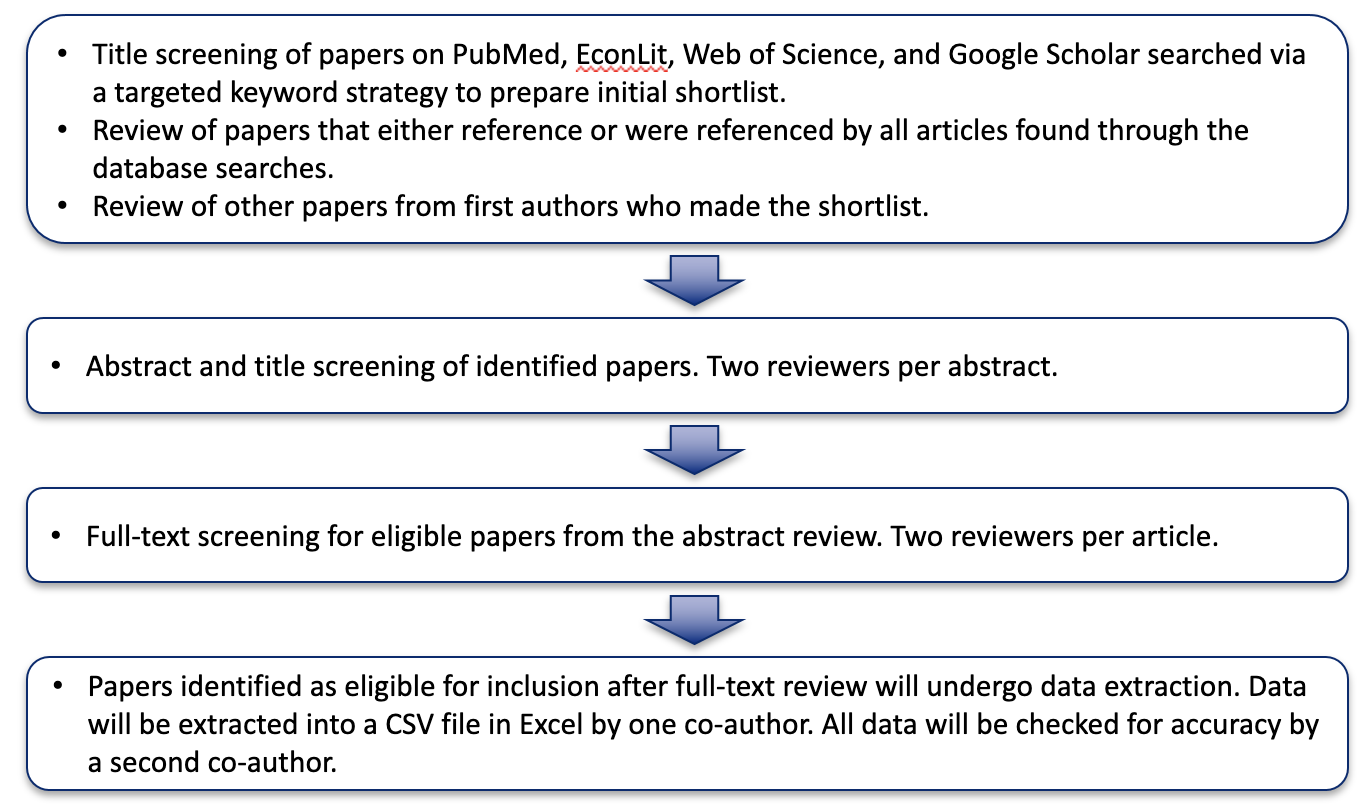


Figure 2. Flow Chart of Search Strategy and Screening Processes.

Table 1. Example Search Strategy

| **Keyword Search** |
| --- |
| (Economic Evaluation) and (Implementation Science) |
| (Cost Effectiveness) and (Implementation Science) |
| (Cost-Effectiveness) and (Implementation Science) |
| (Health Economics) and (Implementation Science) |
|  |
| **MeSH Search** |
| ("Implementation Science"[Mesh]) AND "Health Care Economics and Organizations"[Mesh] |
|  |

## INCLUSION CRITERIA

After a preliminary search and discussion among the team members, the following inclusion criteria were agreed upon.

**Source of Information**: Publications that are accessible publicly and using the Johns Hopkins Academic Library.

**Time Frame**: January 2010 to December 2021

**Language**: English, Spanish, French, Portuguese, and others when possible

**Study Setting:** Studies from low-and-middle-income countries, with inclusion of studies from high-income countries whose methodology and findings are relevant and applicable to low-and-middle-income settings.

**Study population:** All age groups, genders, and populations

**Types of interventions**: Public health interventions utilizing implementation science

**Types of economic evaluations**: All economic evaluations such as costing analysis, cost-effectiveness analysis, cost-utility analysis, benefit incident analysis, budget impact analysis, etc.

**Types of implementation science outcomes**: All outcomes established in implementation science frameworks (e.g. RE-AIM, Proctor, PRECIS-2, etc.)

**Type of studies**: Theoretical and empirical studies, frameworks, methodology papers

**Type of diseases:** Infectious and non-infectious diseases focused on health programs and health policies

**Type of articles**: All (books and documents, clinical trial, meta-analysis, randomized control trial, peer-reviewed journal articles, grey literature, review, and systematic review)

## SOURCE OF EVIDENCE SELECTION

The team will use Mendeley as its reference management software. The following process for filtering sources for evidence selection will be applied by the team:

1. Two team members will review each identified article and determine whether it meets the inclusion criteria.
2. The team members will individually determine which of the shortlisted papers are relevant for conducting in-depth assessment or discussion, to eventually be referenced in the scoping review. (I.e., do the articles discuss the interplay between economic evaluations and implementation outcomes?).
3. A consensus should be reached by all team members while finalizing the sources that meet the inclusion criteria and are considered relevant.
4. If unable to come to a consensus on inclusion of a specific article, the two reviewers will present the article-in-question to the larger team to vote on eligibility.
5. The team will meet routinely to discuss any discrepancies or to make modifications to the selection criteria. The inclusion criteria, or criteria for determining if a paper is relevant, may be revisited if all members of the team feel that certain papers would be useful for the scoping review, and so a modified set of inclusion criteria will enable their use.

## DATA EXTRACTION

Our team will develop a standardized data extraction template. We will capture article details, study details, results and outcomes, and key takeaways from the discussion section of the article. Table 2 describes the categories/subcategories proposed for data extraction.

Table 2. List of Data Extraction Elements from Included Papers

| **Category** | **Subcategory** | **Description** |
| --- | --- | --- |
| Article details | Title | Full title of the article |
|  | Author(s) | List of all authors |
|  | Citation | Citation in APA format |
|  | Objective | Aims or objectives of the study |
|  | Disease | Disease being studied |
|  | Geography | Country or region the article is assessing |
| Study details | Type of Study | Determine whether the study is empirical or theoretical |
|  | Perspective of study | Whether the study captures a patient level, health system or societal perspective. Is the choice in perspective justified? |
|  | Intended Audience | Does the study identify an intended audience? |
|  | Policy Targets | Does the study mention a specific policy, guideline, policy audience, or governing body that the paper is aimed at? |
|  | Study design | What were the methods applied for the study |
|  | Data type | Whether the data was primary or secondary |
|  | Study population | Which population was studied |
|  | Intervention type and comparator group | What Intervention type, comparator, and details of these (e.g., duration of the intervention) |
|  | Type of economic evaluation | Whether it is a costing analysis, cost-effectiveness analysis, cost-utility analysis, benefit incident analysis, or budget impact analysis |
|  | Implementation related costs | Whether implementation related costs are incorporated? |
|  | Linkage to implementation outcomes | Which elements of the implementation process being considered? Are any specific frameworks referenced? |
| Results or outcomes | Results or outcomes | What were the key results from the study? |
|  | Usefulness for Policy | Are the results useful for a policy audience? Is it aimed at real-world implementation or sustainability? |
| Discussion | Key Takeaways and Recommendations | What were the key takeaways or recommendations from the articles? Discuss best techniques and information gaps. |
|  | Best Techniques | What are some of the key methodology techniques identified by the authors? What are the strengths and weaknesses of their approach? |
|  | Information Gaps | Are there any major information gaps identified by the authors? |
|  | Policy Recommendations | Do the authors make any specific policy recommendations based on their results? How do they use the economic and implementation outcomes to frame their recommendation? |

## ANALYSIS OF THE EVIDENCE

Since this is a scoping review and not a systematic review, we will not be conducting risk of bias assessments, performing any data syntheses, or conducting meta-analyses on any specific outcomes. We will develop simple frequency counts of concepts, population, and themes to map what kind of literature is most prevalent. We anticipate that we will be dealing with primarily qualitative results when we conduct an analysis of the extracted data. We will provide a narrative summary of the data we extract, linking the extracted data to our research question and develop a few broad themes from the extracted literature. Looking toward broader implications, we will discuss how the body of extracted evidence could inform economically feasible implementation of health interventions in a real-world LMIC setting. We will evaluate what methodologies already exist for integrating economic evaluation and implementation science outcomes, what recommendations are provided by existing implementation science frameworks, and how these techniques have been utilized in both empiric and theoretical analyses of health interventions. Building on the existing evidence, we will develop standardized operating procedures on how best to collect, analyze, and synthesize economic and implementation data, as well as how to present these results in scientific publications and to stakeholders. We will also discuss gaps in the current literature that may warrant more research in the future.

## PRESENTATION OF THE RESULTS

Tables, charts, flow-charts, conceptual maps and diagrams will be leveraged as appropriate to present the results of the scoping review. As an example, we may use a stacked bar or a regular bar chart to determine either the percentage (share) or the number of articles that could be mapped to a particular theme identified during the process of our review. Such visual tools will make it easier for the reader to follow the review.

## DISCUSSION AND DISSEMINATION

It is expected that the findings from this scoping review will provide a thorough examination of the available evidence on the synthesis of implementation outcomes and economic evaluations in LMICs. The review will provide a full summary of the existing methodologies and techniques used to integrate the two fields, explore the strengths and weaknesses of different methods, and propose new and alternate approaches to integrating economic evaluation and implementation science outcomes. It will provide methodologies and a framework for future economic evaluations in the implementation science realm, building on existing frameworks and literature, to generate standardized techniques for data collection and analysis for researchers and policymakers to use in their economic decision-making. The findings from this scoping review will be disseminated through publication in a peer-reviewed journal and through presentations at conferences.

This protocol presents the complete methodology for a scoping review on the integration of economic evaluation and implementation science health interventions in low-and-middle income countries and outlines how this information will be used to develop standardized methodology for the field going forward.

# Proposed Contributors

*Akash Malhotra. [Amalhot7@jh.edu](mailto:Amalhot7@jh.edu). Johns Hopkins Bloomberg School of Public Health, Baltimore, MD.

*Ryan R. Thompson. [Rthomp67@jh.edu](mailto:Rthomp67@jh.edu). Johns Hopkins Bloomberg School of Public Health, Baltimore, MD.

David W. Dowdy. Johns Hopkins Bloomberg School of Public Health, Baltimore, MD.

Hojoon Sohn. Seoul National University College of Medicine. Seoul, KR.

Drew Cameron. Yale School of Public Health, New Haven, CT, USA.

Abigail Barker. Washington University in Saint Louis, Saint Louis, MO, USA.

William Effah. Washington University in Saint Louis, Saint Louis, MO, USA.

Victor G. Davila-Roman. Washington University in Saint Louis, Saint Louis, MO, USA.

David Watkins. University of Washington, Seattle, WA, USA

Michael Laxy. Technical University of Munich. Munich, DE.

Jane Phiri. University of Witwatersrand, Johannesburg, SA.

Faith Kagoya. Infectious Diseases Research Collaboration. Kampala, UG.

Mosepele Mosepele. University of Botswana, Gaborone, BW.

Peter Mbewe. Center for Infectious Disease Research in Zambia. Lusaka, ZM.

Felix Masiye. University of Zambia, Lusaka, ZM.

Jairos Sambo. Cavendish University Zambia, Lusaka, ZM.

*Corresponding Authors

# Funding Information

This scoping review is funded by the National Heart, Lung, and Blood Institute (U24HL154426).

# References

1. Husereau D, Drummond M, Petrou S, et al. Consolidated Health Economic Evaluation Reporting Standards (CHEERS) statement. *BMJ*. 2013;346(March):1-6. doi:10.1136/bmj.f1049

2. Husereau D, Drummond M, Augustovski F, et al. Consolidated Health Economic Evaluation Reporting Standards 2022 (CHEERS 2022) statement: updated reporting guidance for health economic evaluations. *MDM Policy Pract*. 2022;7(1):238146832110610. doi:10.1177/23814683211061097

3. Wilkinson T, Sculpher MJ, Claxton K, et al. The International Decision Support Initiative Reference Case for Economic Evaluation: An Aid to Thought. *Value Heal*. 2016;19(8):921-928. doi:10.1016/j.jval.2016.04.015

4. Institute for Clinical and Economic Review. ICER's Reference Case for Economic Evaluations: Principles and Rationale. Accessed 24 March 2022. Available at: https://icer.org/wp-content/uploads/2020/10/ICER_Reference_Case_013120.pdf

5. NICE International. Methods for Economic Evaluation Project: Final Report. *Bill Melinda Gates Found Methods Econ Eval Proj*. 2014;(January):30-33. http://www.idsihealth.org/wp-content/uploads/2015/01/MEEP-report.pdf

6. Vassall A, Sweeney S, Kahn JG, et al. Reference Case for Estimating the Costs of Global Health Services and Interventions. *Global Health Costing Consortium*. Accessed 25 March 2022. Available at: https://researchonline.lshtm.ac.uk/id/eprint/4653001/1/vassall_etal_2018_reference_case_for_estimating_costs_global_health_services.pdf

7. Glasgow RE, Vogt TM, Boles SM. Evaluating the public health impact of health promotion interventions: The RE-AIM framework. *Am J Public Health*. 1999;89(9):1322-1327. doi:10.2105/AJPH.89.9.1322

8. Proctor E, Silmere H, Raghavan R, et al. Outcomes for implementation research: Conceptual distinctions, measurement challenges, and research agenda. *Adm Policy Ment Heal Ment Heal Serv Res*. 2011;38(2):65-76. doi:10.1007/s10488-010-0319-7

9. Aromataris E, Muzz Z (Editors). JBI Manual for Evidence Synthesis. JBI, 2020. Accessed 24 March 2022. Available at: https://jbi-global-wiki.refined.site/space/MANUAL

10. Tricco AC, Lillie E, Zarin W, et al. PRISMA extension for scoping reviews (PRISMA-ScR): Checklist and explanation. *Ann Intern Med*. 2018;169(7):467-473. doi:10.7326/M18-0850

11. CADTH. Grey Matters: a practical tool for searching health-related grey literature | CADTH.ca. *Cadth*. Accessed 25 March 2022. Available at: https://www.cadth.ca/resources/finding-evidence/grey-matters
